# Hypertension and type 2 diabetes: a cross-sectional study in Morocco (EPIDIAM Study)

**Published:** 2012-03-20

**Authors:** Mohamed Berraho, Youness El Achhab, Abdelilah Benslimane, Karima EL Rhazi, Mohamed Chikri, Chakib Nejjari

**Affiliations:** 1 Laboratoire d’Epidémiologie, Recherche Clinique et Santé Communautaire; Faculté de Médecine et de Pharmacie, Fès, Maroc; 2 Laboratoire de Biochimie, Faculté de Médecine et de Pharmacie, Fès, Maroc

**Keywords:** Hypertension, diabetes, Morocco, risk factors, Anthropometric measurements, cross-sectional survey

## Abstract

**Background:**

In Morocco, there are no studies that focused on the hypertension and its associated risk factors through patients with type 2 diabetes. Different findings show that the frequency of type 2 diabetes has risen rapidly in Morocco. The main objective of this study was to assess the prevalence of hypertension and its associated risk factors among a group of patients with type 2 diabetes and to examine the level of control of hypertension among type 2 diabetic patients with hypertension.

**Methods:**

A cross-sectional study was carried out on 525 type 2 diabetics in three Moroccan regions. The structured questionnaire was used to gather information on sociodemographic variables, history of hypertension, use of anti-hypertensive medications and duration of diabetes. Anthropometric measurements including weight and height were measured by trained staff. Blood pressure was measured using standardized sphygmomanometers.

**Results:**

The prevalence of hypertension was 70.4%. The logistic regression indicated that hypertension was positively associated with age (p<10–4), BMI (p<0.0002) and duration of diabetes (p)

**Conclusion:**

Hypertension is a common co-morbidity among Moroccan diabetic patients with high rate of ignorance of hypertension among study subjects. The focus must be on patients and family education, counseling and behavioral interventions designed to modify lifestyle such as increasing physical activity and adopting recommended dietary changes, as well as compliance with medications.

## Background

Type 2 diabetes is estimated to affect over 150 million people world-wide [1]. This prevalence is increasing rapidly, partly through changes in case ascertainment and diagnostic criteria, but mainly through lifestyle changes in countries which know a fast development [1]. Type 2 diabetes is also associated with an increased risk of premature death due to cardiovascular disease (CVD), stroke, and renal disease [2]. Hypertension is a major risk factor for cardiovascular disease, stroke and ischemic heart disease. Therefore, this factor represents one of the most preventable causes of morbidity and premature mortality in developed as well as developing countries [3]. Hypertension and diabetes frequently coexist. The frequency of hypertension in diabetic population is almost twice as compared to non-diabetic general population [4].

There is a considerable evidence for an increased prevalence of hypertension in diabetic persons [5]. The prevalence rate of hypertension among type 2 diabetics is higher than that of age and sex-matched patients without diabetes, ranging between 32% and 82% [6–11].

The coexistence of hypertension and type 2 diabetes is a major contributor to the development and progression of macrovascular and microvascular complications in people with diabetes compared to the general population [3,12–14].

Both hypertension and diabetes predispose to the development of CVD and renal disease [15,16]. The presence of hypertension in diabetic patients substantially increases the risks of coronary heart disease, stroke, nephropathy and retinopathy [17–19]. Indeed, when hypertension coexists with diabetes, the risk of CVD is increased by 75%, which further contributes to the overall morbidity and mortality of an already high risk population [17,20].

In Morocco, there are no studies that have focused on hypertension and its associated risk factors through patients with type 2 diabetes. The data of the Ministry of Health in Morocco reports an increase in consultations related to diabetes [21]. The national study, on cardiovascular risk factors, conducted during the year 2000 on a Moroccan representative sample aged 20 years old and over showed that the prevalence of diabetes was 6.6%, the prevalence of hypertension was 33.6% (30.2% for men and 37.0% for women) and the prevalence of cardiovascular risk factors was high within the Moroccan population [22].

The main objective of this paper is to assess the prevalence of hypertension and its associated risk factors among a group of patients with type 2 diabetes and to examine the level of control of hypertension among type 2 diabetic patients with hypertension.

## Methods

### Design and sample

A cross-sectional study design was used to estimate the frequency rates of hypertension among a population of type 2 diabetic patients in three Moroccans regions (Fez, Sale and Taounate), in partnership with the delegation of the Ministry of Health and associations working in social and health domains. The investigation was led during the period from February to July 2006 in centers of health assuring general medicine consultations. People of every region were informed two weeks before days by local authorities. Volunteers and accepting participants in the study were recruited.

### Questionnaire

A structured questionnaire was used to gather information on sociodemographic variables (gender, age and level of education), smoking status, history of hypertension, use of anti-hypertensive medications and duration of diabetes. Anthropometric measurements including weight and height were measured by trained staff. Body mass index (BMI) was obtained by dividing the weight in kilograms by the square of the height in meters. BMI was categorized as underweight < 18.5 kg/m^2^, normal if 18.5-24.9 kg/m^2^, overweight if 25-29.9 kg/m^2^, and obesity if = 30 kg/m^2^ [23]. Blood pressure was measured using standardized sphygmomanometers. A trained nurse performed the procedures while the subject was in a sitting position with the arm at the level of the heart and after 5 minutes rest. Two blood pressure readings were taken from each patient and the average reading of both was used in this study. The patient was labeled as having hypertension if systolic blood pressure =140 mm Hg or diastolic blood pressure =90 mm Hg, or if the patient was on antihypertensive medications [24]. Diabetic patients who are already known to have hypertension were considered to get uncontrolled hypertension if systolic blood pressure =140 mm Hg or diastolic blood pressure =90 mm Hg, or if the patient was on antihypertensive medications [24].

### Analysis

Data analysis was performed using the Epi-info 2000. Frequency distributions and chi-square statistics were used for categorical variables. Logistic regression analyses were performed to assess the independent effect of age, gender, education level, BMI, smoking status, physical activities and duration of diabetes on hypertension. The 95% confidence interval was calculated using the standard error of the regression coefficient.

## Results

As shown in Table 1, of 525 diabetic participants 68.7% were females, 47.1% were >60 years old and 77.3% were illiterate. The half of sample (50.3%) had been suffering from overt diabetes for a period less than 5 years, 42.7% have overweight and 31.2% were obese, 2.7% were current smokers and 5.9% were former smokers. The prevalence rate of hypertension was 70.4%. In Table 1, we present the sociodemographic characteristics of study population according to hypertension status. The prevalence among men was similar to that among women (P=0.31). The rate of hypertension increased with age (P=0.001). Illiterate people were found to be at higher risk of hypertension compared to those with a high school or college education (P<10^−3^). Obese and underweight subjects had significantly (P=0.01) got higher rate of hypertension (77.4% and 80.0% respectively) than overweight (70.9%) and normal weight (60.6%) groups. Contrary to our expectations, non-smokers in this study had a higher rate of hypertension than smokers (P<10^−2^). Patients without physical activity were found to be at higher risk of hypertension compared to those with physical activity (P

**Table 1:** Sociodemographic characteristics of study population according to hypertension status (N=525). EPIDIAM Study - Morocco 2006.

	Total	Hypertension		P
		Yes	No	
	N (%)	N (%)	N (%)	
**Gender**				
Female	361 (68.7)	260 (72.0)	101 (28.0)	0.31
Male	134(31.3)	111 (67.7)	53 (32.3)	
**Age** (years)				
<50	127 (24.2)	54 (42.5)	73 (57.5)	<10^−3^
50–60	151 (28.8)	110 (72.9)	41 (27.1)	
≥60	247 (47.1)	207 (83.8)	40 (16.2)	
**Educational level**				
Uneducated	406 (77.3)	305 (75.1)	101 (24.9)	<10^−3^
Primary education	66 (12.6)	35 (53.0)	31 (47.0)	
Secondary education	40 (7.6)	25 (62.5)	15 (37.5)	
High education	13 (2.5)	6 (46.2)	7 (53.8)	
**BMI** (Kg/m^2^)				
<18.5	10 (1.9)	8 (80.0)	50 (20.0)	0.01
18.5–25	127 (24.2)	77 (60.6)	2 (39.4)	
25–30	224 (42.7)	159 (70.9)	65 (29.1)	
30–35	164 (31.2)	127 (77.4)	37 (22.6)	
**Smoking status**				
Current Smokers	14 (8.5)	5 (35.7)	9 (64.3)	<10^−2^
Former smokers	31 (18.9)	18 (58.1)	13 (41.9)	
Non smokers	480 (72.6)	348 (72.5)	132 (27.5)	
**Physical activities**				
More than 3 times / week	36 (6.7)	18 (50.0)	18 (50.0)	<0.01
1 to 2 times / week	55 (10.5)	37 (67.3)	18 (32.7)	
No physical activity	434 (82.7)	316 (72.8)	118 (27.2)	
**Duration of diabetes**				
<5 years	264 (50.3)	168 (63.6)	96 (26.4)	<10^−2^
5–10 years	127 (24.2)	102 (80.3)	25 (19.7)	
≥10 years	134 (25.5)	101 (75.4)	33 (24.6)	

* Smoking status in male only (no woman was current or former smoker in our study)

In the multivariate analysis (Table 2), there was a positively and statistically significant association between hypertension and the variables of age, BMI and duration of diabetes. But no significant association was found with level of education or physical activity. Compared to the age group < 50 years, the risk of hypertension increased by 3.98 times among the 50-59 age group (P<10^−4^) and by 7.26 times among the age group 60 years old and up (P<10^−4^). Compared to the normal BMI group, the risk of hypertension increased by 3.09 among the obese group (P

**Table 2:** Adjusted odds ratio (OR) for prevalence of hypertension by selected variables in multivariate analysis (N=525). EPIDIAM Study -Morocco 2006

	OR	IC_OR_	*P*	*P*_*t*_
**Gender** (Female Vs Male)	0.96	0.55-1.67		0.88
**Age** (years)				
<50	**1**	**–**	**–**	
50–60	3.98	2.31**–**6.85	<10^−4^	<10^−4^
≥60	7.26	4.20**–**12.54	<10^−4^	
**Education level**				
Uneducated	1.62	0.43**–**5.89	0.47	
Primary education	0.72	0.18**–**2.84	0.63	0.07
Secondary education	1.67	0.39**–**7.07	0.48	
High education	**1**	**–**	**–**	
**BMI** (Kg/m2)				
<18.5	4.06	0.59-28.10	0.16	
18.5–25	**1**	**–**	**–**	0.002
25–30	1.73	1.03**–**2.91	0.04	
30–35	3.09	1.72**–**5.56	0.0002	
**Smoking status**				
Current Smokers	0.22	0.06-0.81	0.02	
Former smokers	0.63	0.24-1.66	0.35	0.06
Non smokers	**1**	**–**	**–**	
**physical activity**				
More than 3 times / week	**1**	**–**	**–**	
1 to 2 times / week	2.53	0.85-6.45	0.10	0.17
No physical activity	2.11	0.94 4.74	0.07	
**Duration of diabetes**				
< 5 years	1	**–**	**–**	
5–10 years	2.57	1.45**–**4.55	0.001	0.004
≥10 years	1.49	0.87**–**2.54	0.14	

OR: Odd ratio; IC: Confidence Interval

Of 371 hypertensive patients 38.8% were not aware of having hypertension at the time of the study. Almost the majority (82.8%) of patients who were aware of having hypertension had failed to keep their blood pressure levels under control. As indicated in Table 3, no significant association was found between the rate of uncontrolled hypertension and the variables of gender, level of education, BMI, physical activity and duration of diabetes. But there was a significant association between the rate of uncontrolled hypertension and age; the rate of uncontrolled hypertension was conversely proportional to age (Table 3).

**Table 3:** Chi-square distribution of controlled and uncontrolled hypertension among previously diagnosed hypertensive by selected variables (n=227). EPIDIAM Study - Morocco 2006

	Uncontrolled Hypertension (N=188)		Controlled Hypertension (N=39)		P
	n	(%)	n	(%)	
**Gender**					
Female	33	(19.9)	133	(80.1)	0.08
Male	6	(9.8)	55	(90.2)	
**Age** (years)					
<50	9	(36.0)	16	(64.0)	
50–60	13	(24.5)	40	(75.5)	0.003
≥60	17	(11.4)	132	(88.6)	
**Education level**					
Uneducated	29	(15.3)	160	(84.7)	
Primary education	4	(22.2)	14	(77.8)	–
Secondary education	4	(25.0)	12	(75.0)	
High education	2	(50.0)	2	(50.0)	
**BMI** (Kg/m2)					
<18.5	3	(50.0)	3	(50.0)	
18.5–25	7	(16.7)	35	(83.3)	–
25–30	15	(15.6)	81	(84.4)	
30–35	14	(16.9)	69	(83.1)	
**Smoking status**					
Current Smokers	0	(0.0)	1	(100)	
Former smokers	1	(7.1)	13	(92.9)	–
Non smokers	38	(17.9)	174	(82.1)	
**Physical activity**					
More than 3 times / week	2	(33.3)	4	(66.7)	
1 to 2 times / week	9	(42.9)	12	(57.1)	–
No physical activity	28	(14.0)	172	(86.0)	
**Duration of diabetes**					
<5 years	13	(15.5)	71	(84.5)	
5–10 years	11	(18.0)	50	(82.0)	0.87
≥10 years	15	(38.5)	67	(81.7)	

## Discussion

The present study reports that hypertension is a very common co-morbidity among Moroccan patients with type 2 diabetes. Therefore, we found a positive association of hypertension with age, BMI and duration of diabetes. Of those who had been diagnosed hypertensive, 38.8% were not aware of their hypertension at the time of the study. Of 227 type 2 diabetic patients who were aware of having hypertension we found a lack (17.2%) of adequately controlled blood pressure.

Hypertension is a common problem for people with diabetes. Compared to Arab population, the prevalence rate of hypertension reported in this study (70.4%) among patients with type 2 diabetes is comparable to the 64.5% rate reported in Qatari diabetics [25] and 72.4% rate reported in Jordanian diabetics [26]. In other Arab populations, the prevalence rate of hypertension is moderate: 53% in Saudi diabetics [27], 44% in Omani diabetics [28] and 38% in Bahraini diabetics [29].

Compared to other populations, the rate of hypertension among diabetics in our study is comparable to the 74%, 74.4% and 73% rates of hypertension reported in UK Caucasians [8], Italian [9] and Spanish [10] populations, respectively. This prevalence is lower than the 82% prevalence rate reported about Afro-Caribbean individuals living in UK [11] and much higher than the 32% and 39% rates reported among diabetics in the Turkish [6] and Taiwanese [7] populations, respectively.

The relatively higher rate of hypertension reported in this study is related to the fact that most diabetic patients were aged 60 years old and over. The explanation for differences in frequency by each country could be due to different methods of surveillance, differences in definitions of hypertension, population characteristics and ethnic variations [11].

Hypertension amongst type 2 diabetics was associated with age; this association agrees with research literatures and with the findings of other studies [30–34].

Our study also showed that obese and overweight patients have a higher risk of hypertension than ones with normal BMI, this association is in agreement with research literatures and with the findings of other studies [34]. In addition, the coexistence of diabetes, hypertension and obesity or overweight increases the risk of cardiovascular complications and other morbidities [36,37].

Our data indicated that hypertension is associated with the duration of diabetes. Duration of diabetes is positively associated with the severity of macro- and micro-vascular complications, both of which contribute to the development of renal and/or atherosclerotic hypertension [3,12–14].

Our expectations showed that there is no significant association between hypertension and smoking. This finding is in contrary with findings reported in the research literature [38]. This situation may be explained by the lack of smokers in our study and the minority of male participants.

The frequency of unknown hypertension status in this diabetic population was high (38.8%). This result draws attention to the importance of monitoring and control of blood pressure in diabetics and the importance of monitoring education for a diabetic patient.

The majority of patients, in our study, with previously diagnosed hypertension still had uncontrolled hypertension. This finding is consistent with findings reported in other studies [39, 40]. This may be due to the patient’s underestimation of the potential complications of hypertension, non-compliance, absence of effective health education programs, low level of education and/or low socioeconomic levels. Other studies planned in order to explain this situation are necessary. Indeed, a very high percentage of our population is illiterate. Poor education is usually associated with low income and may contribute to the high rate of uncontrolled hypertension. Anti-hypertensive treatments are expensive and these patients may not be able to afford their costs.

The main limit of our study is that we did not get a representative sample. Thus, more studies using thorough sampling method are needed. In fact, in Morocco there is no registry of diabetics. What we do not allow a comparison between our sample and the population of Moroccan diabetics. A data from a study on the epidemiology of hypertension and other cardiovascular risk factors, conducted in 2000 on a representative sample of the Moroccan population showed that among 1802 participants 6.6% (n=116) were diabetic and among those with diabetes 66.4% were hypertensive [22]. This result is slightly lower than our result (70.4%). Despite this limitation, our study is the only one in Morocco done on a large sample, which was specifically interested in the problem of hypertension in diabetic patients and reported very important information on the epidemiology of hypertension in Moroccan diabetics.

## Conclusion

The prevalence of hypertension, the frequencies of undiagnosed hypertension and uncontrolled hypertension among Moroccan patients with type 2 diabetes were very high. The focus must be on patients and family education, counseling and behavioral interventions designed to modify lifestyle such as increasing physical activity and adopting recommended dietary changes, as well as compliance with medications. Thus, these results can serve as a wake-up appeal for more researches based on health care needs of these regions.
